# Supplementary material for: A genetic screen in enteroendocrine cells reveals mechanisms that control protein sensing and GLP-1 release
Source: bioRxiv. 2026 Jan 6:2025.11.30.691441. Preprint. [Version 2] doi: 10.64898/2025.11.30.691441 (PMC12802397; doi:10.64898/2025.11.30.691441)
Supplement: Supplement 1 [file NIHPP2025.11.30.691441v2-supplement-1.pdf]

bioRxiv preprint doi: https://doi.org/10.64898/2025.11.30.691441; this version posted January 6, 2026. The copyright holder for this preprint (which was not certified by peer review) is the author/funder, who has granted bioRxiv a license to display the preprint in perpetuity. It is made available under aCC-BY 4.0 International license.

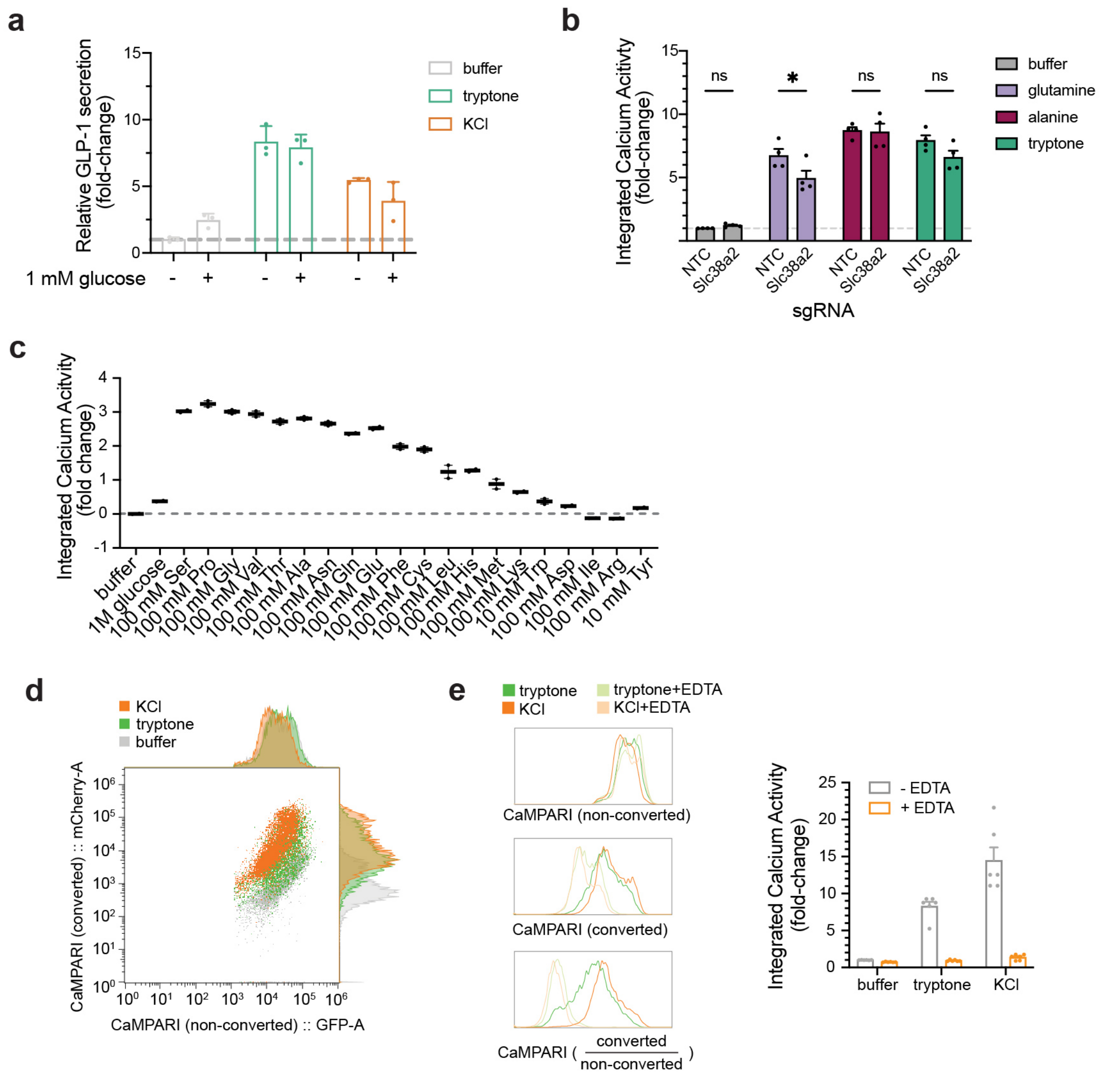

Supplemental Figure 1.
a, GLP-1 secretion in STC-1 does not depend on addtion of glucose.
b, Integrated calcium activity for tryptone and Slc38a2 substrates after NTC or Slc38a2 KD.
c, Integrated calcium activity (median CaMPARI photoconversion ratio) for individual amino acids in STC-1.
d, Raw flow cytometry data for Figure 1h.
e, EDTA completely inhibits stimulated calcium acitvity in STC-1. d, Histogram of CaMPARI activity under indicated conditions. e, Median CaMPARI photoconversion ratio from each flow cytometry session is plotted.

bioRxiv preprint doi: https://doi.org/10.64898/2025.11.30.691441; this version posted January 6, 2026. The copyright holder for this preprint (which was not certified by peer review) is the author/funder, who has granted bioRxiv a license to display the preprint in perpetuity. It is made available under aCC-BY 4.0 International license.

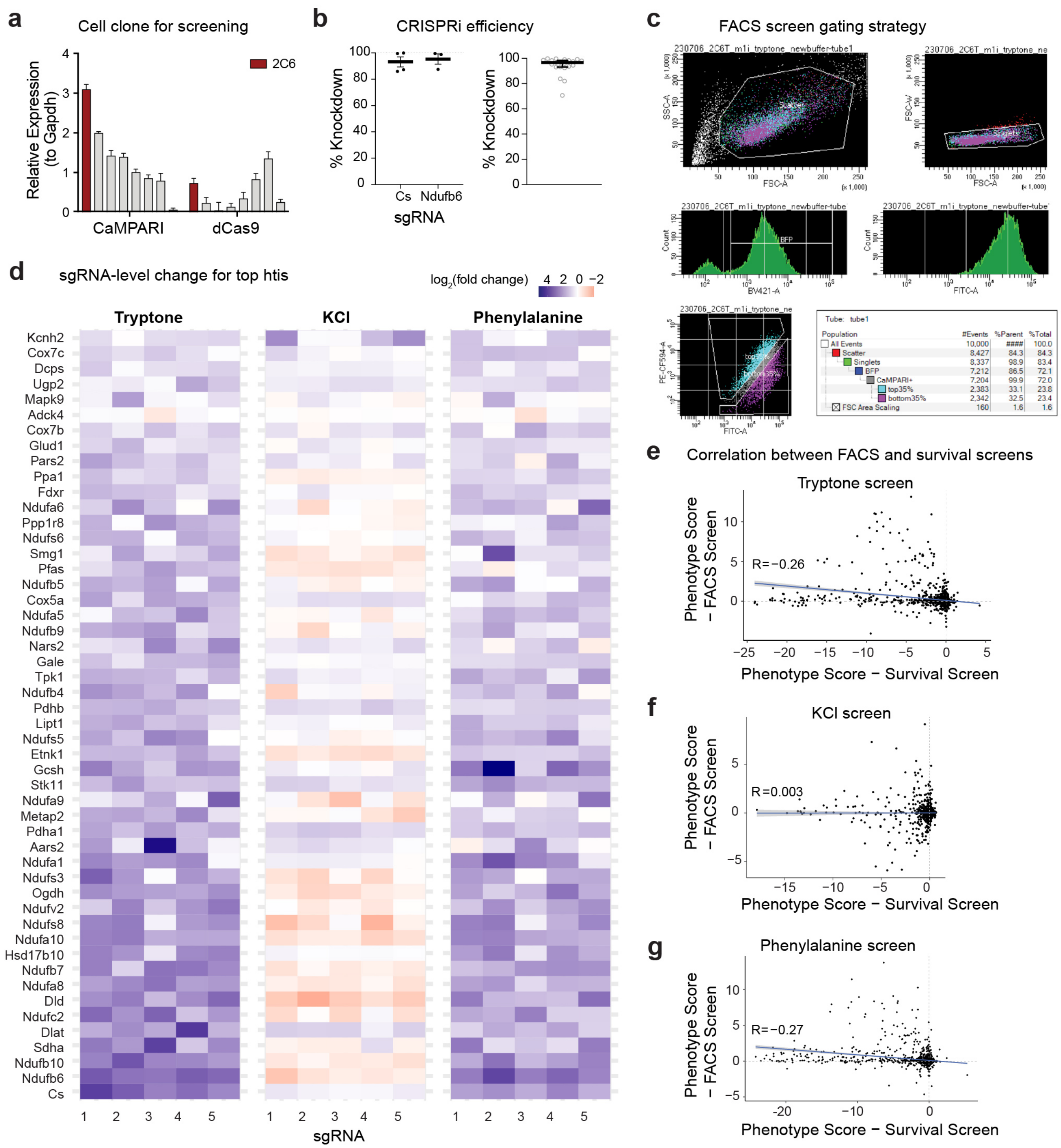

Supplemental Figure 2.
a, Expression levels of CaMPARI and ZIM3-KRAB-dCas9 in each isolated single clones. Clone 2C6 is selected for all the CaMPARI screens and validation shown in this manuscript.
b, CRISPRi efficiency by qPCR. Left, Knockdown efficiency for two candidate genes shown in Figure 4. Right, Knockdown efficiency for all tested target genes.
c, FACS screen gating strategy. Top and bottom 35% of CaMPARI photoconversion ratio (red/green) was collected.
d, Heatmap showing $\log_2$(fold change) for each individual sgRNA (5 per gene) targeting the top 50 hits from the tryptone screen.
e–g, Phenotype scores for all library genes, comparing FACS screen with interal survival screen control. e, Tryptone screen. f, KCl screen. g, Phenylalanine screen. Pearson correlation coefficient is shown on the plot.

bioRxiv preprint doi: https://doi.org/10.64898/2025.11.30.691441; this version posted January 6, 2026. The copyright holder for this preprint (which was not certified by peer review) is the author/funder, who has granted bioRxiv a license to display the preprint in perpetuity. It is made available under aCC-BY 4.0 International license.

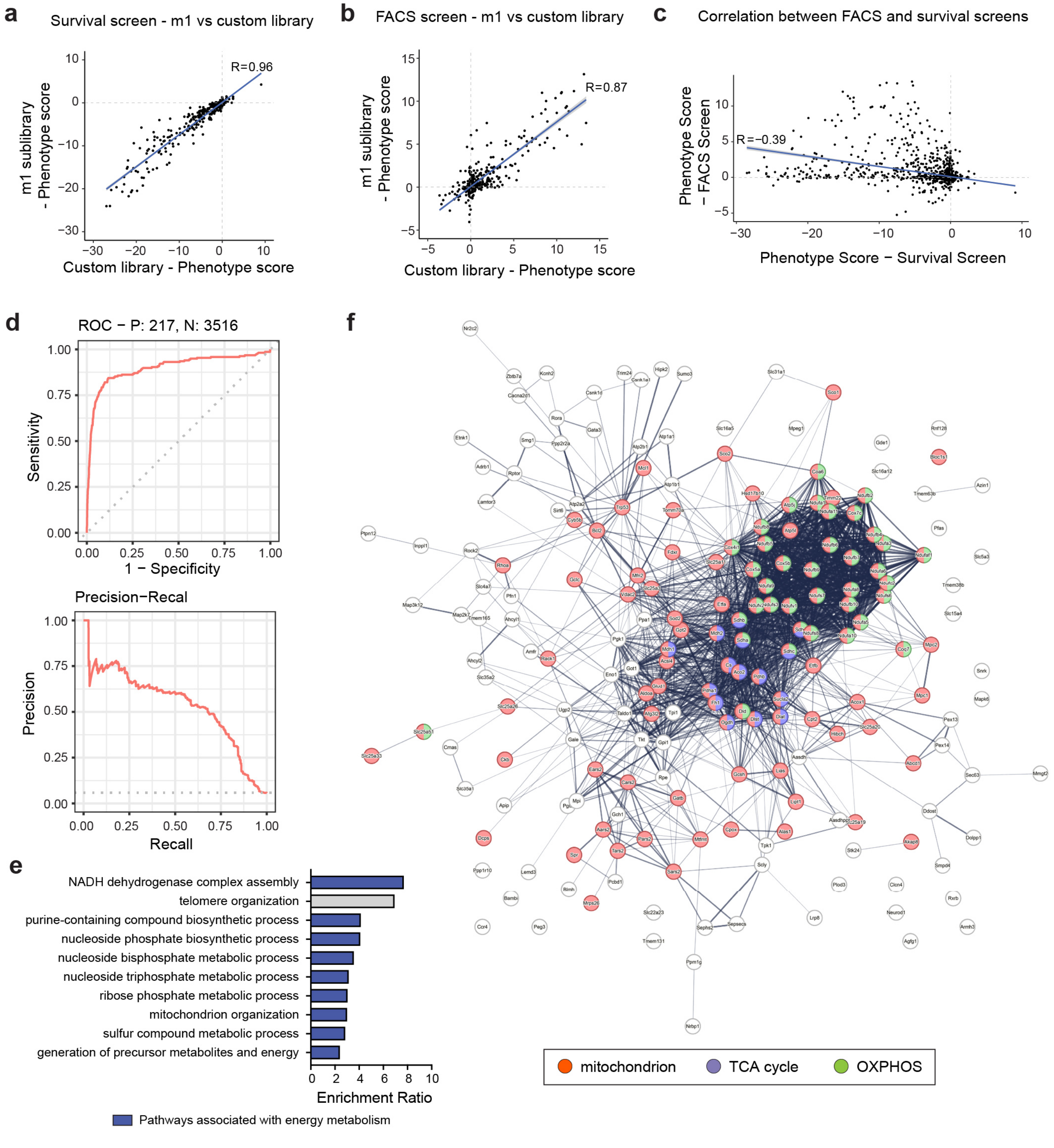

Supplemental Figure 3.
a–b, Correlation of phenotype score between m1 subpoole library screen vs. custom library screen. All hits present in both libraries are shown. a, Comparison between two survival screens. b, Comparison between two FACS screens.
c, Correlation of phenotype score between FACS screen and survival screen for the large-scale custom library screen.
d, Precision-recall analysis of survival screen showed reliable detection of essential genes.
e, Top 10 most enriched pathways for all positive hits from the custom library screen. pathways associated with energy metabolism are highlighted.
f, Functional protein-protein network analysis by STRING (as in Figgure 3d) labeled with all gene names. Genes involved in TCA cycle (GO:0006099) and OXPHOS (GO:0006119) are also highlighted.

bioRxiv preprint doi: https://doi.org/10.64898/2025.11.30.691441; this version posted January 6, 2026. The copyright holder for this preprint (which was not certified by peer review) is the author/funder, who has granted bioRxiv a license to display the preprint in perpetuity. It is made available under aCC-BY 4.0 International license.

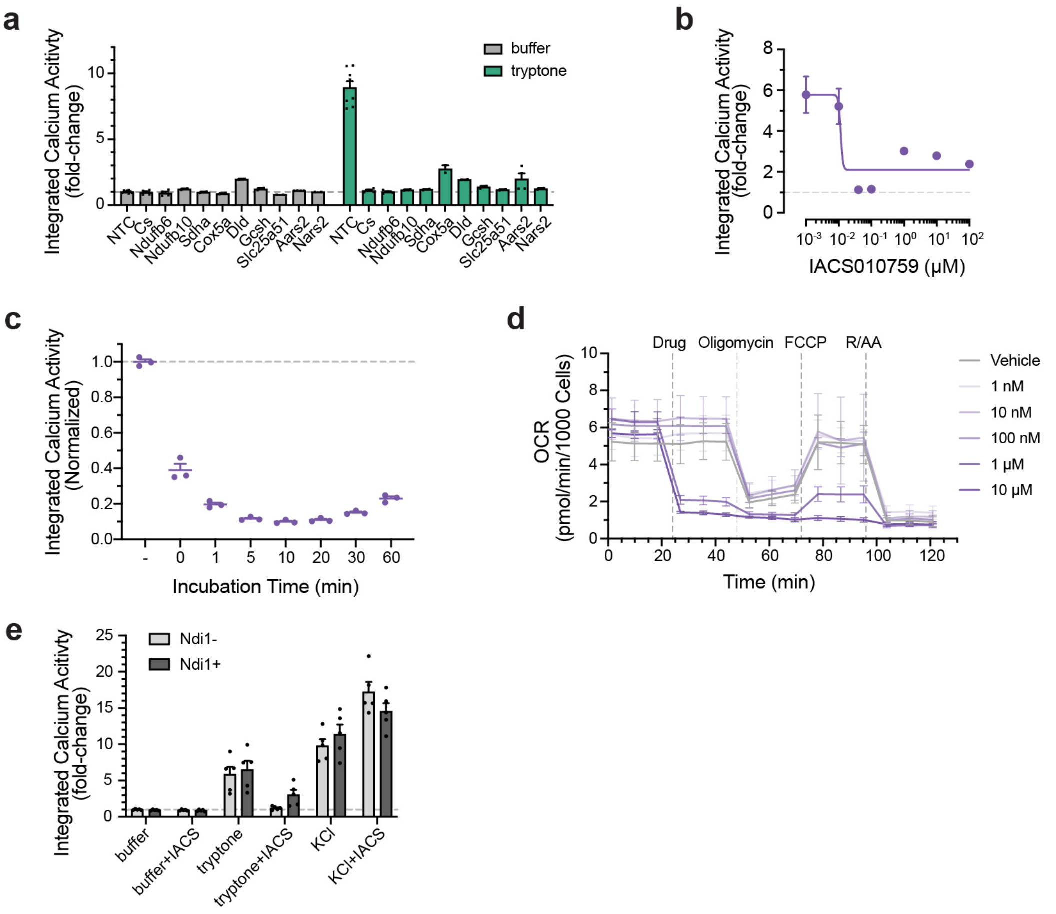

Supplemental Figure 4.

a, Validation of additional hits from the screen by CaMPARI.

b, Dose response curve for the inhibitory effect of IACS010759 on tryptone-induced calcium activity.

c, Inhibition of tryptone-induced calcium activity by IACS010759 after pretreatment of increasing length of time.

d, Oxygen comsuption rate after vehicle or different concentrations of IACS010759 measured by Seahorse Analyzer.

e, Integrated calcium activity with or without stable expression of yeast Ndi, the OXPHOS complex I equivalent.

*P < 0.05; **P < 0.01; ***P < 0.001; ****P < 0.0001.

bioRxiv preprint doi: https://doi.org/10.64898/2025.11.30.691441; this version posted January 6, 2026. The copyright holder for this preprint (which was not certified by peer review) is the author/funder, who has granted bioRxiv a license to display the preprint in perpetuity. It is made available under aCC-BY 4.0 International license.

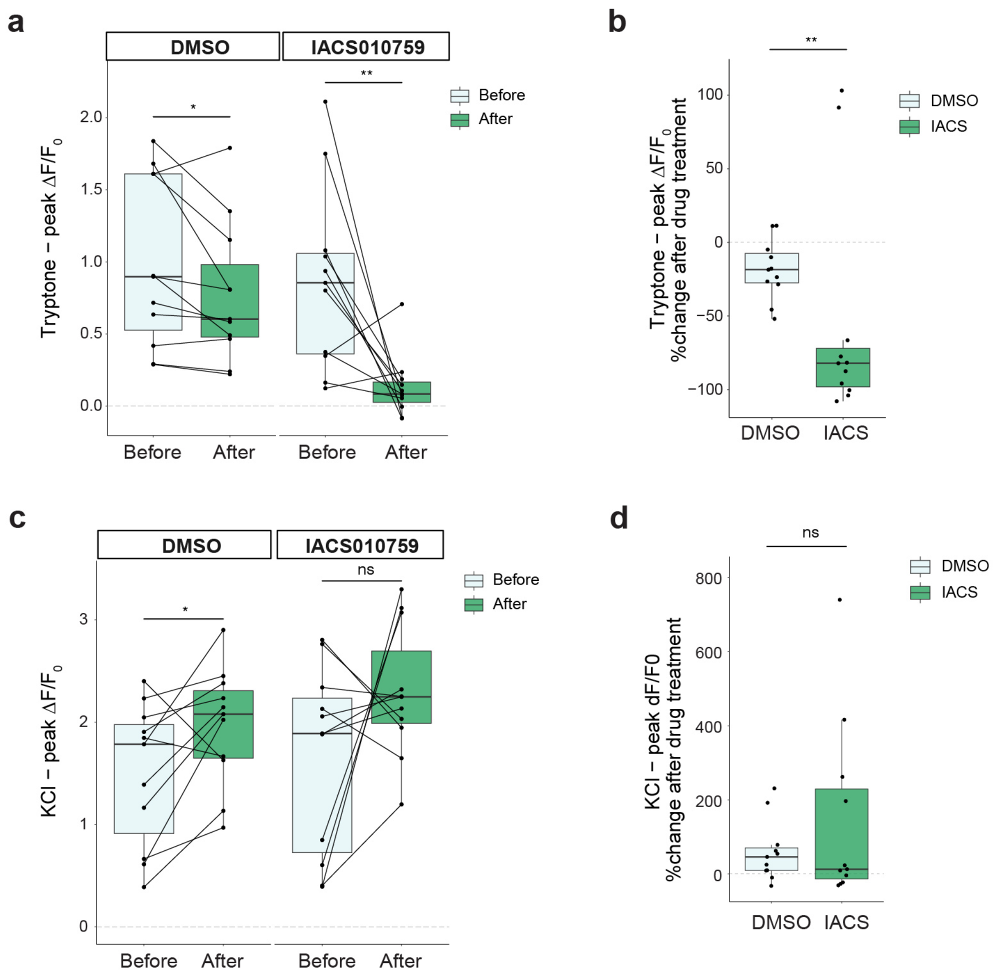

Supplemental Figure 5.
a–b, Quantification of calcium activity in primary EECs before and after treatment. Pairwise comparison of same cells is shown. a, Peak $\Delta F/F_0$ for tryptone. b, Percent of change after trytpone treatment for peak $\Delta F/F_0$.
c–d, Quantification of calcium activity in primary EECs before and after treatment. Pairwise comparison of same cells is shown. c, Peak $\Delta F/F_0$ for KCl. d, Percent of change after KCl treatment for peak $\Delta F/F_0$.

*P < 0.05; ***P < 0.001; ****P < 0.0001.
